# Mesenchymal Stem Cells for Regenerative Therapy: Optimization of Cell Preparation Protocols

**DOI:** 10.1155/2014/951512

**Published:** 2014-01-06

**Authors:** Chiho Ikebe, Ken Suzuki

**Affiliations:** William Harvey Research Institute, Barts and The London School of Medicine and Dentistry, Queen Mary University of London, Charterhouse Square, London EC1M 6BQ, UK

## Abstract

Administration of bone marrow-derived mesenchymal stem cells (MSCs) is an innovative approach for the treatment of a range of diseases that are not curable by current therapies including heart failure. A number of clinical trials have been completed and many others are ongoing; more than 2,000 patients worldwide have been administered with culture-expanded allogeneic or autologous MSCs for the treatment of various diseases, showing feasibility and safety (and some efficacy) of this approach. However, protocols for isolation and expansion of donor MSCs vary widely between these trials, which could affect the efficacy of the therapy. It is therefore important to develop international standards of MSC production, which should be evidence-based, regulatory authority-compliant, of good medical practice grade, cost-effective, and clinically practical, so that this innovative approach becomes an established widely adopted treatment. This review article summarizes protocols to isolate and expand bone marrow-derived MSCs in 47 recent clinical trials of MSC-based therapy, which were published after 2007 onwards and provided sufficient methodological information. Identified issues and possible solutions associated with the MSC production methods, including materials and protocols for isolation and expansion, are discussed with reference to relevant experimental evidence with aim of future clinical success of MSC-based therapy.

## 1. Introduction 

Recent research has extensively shown the potential of bone marrow- (BM-) derived mesenchymal stem cells (MSCs) for regenerative therapies in various organs including the heart [1]. The effects from this approach are dependent on their potency of secretion of beneficial cytokines and growth factors for tissue repair/regeneration and immunomodulation and/or their differentiation for regenerating damaged organs [2]. Since the first clinical trial of BMC injection in 1995 [3], more than 2,000 patients have been administered with allogeneic or autologous MSCs for the treatment of various diseases, including graft-versus-host disease, hematologic malignancies, cardiovascular diseases, neurologic diseases, autoimmune diseases, organ transplantation, refractory wounds, and bone/cartilage defects [4]. More than 200 clinical trials of MSC-based therapy, completed or ongoing, have been listed on the website of the United States National Institute of Health (http://www.ClinicalTrial.gov/) as of July 2013. The cells used are, strictly speaking, mesenchymal stromal cells, which include MSCs and other cells; but, in most cases they are simply referred to as MSCs. Previous pre-clinical studies and clinical trials have shown feasibility and safety of MSC-based therapy; however, the therapeutic effects observed in clinical trials to date appear to be inconsistent and remain inconclusive [5].

MSCs were first described in 1976 by Friedenstein and colleagues [6] and are more recently defined by The International Society of Cellular Therapy based on three cellular properties: (1) adherence to plastic, (2) positive expression of CD105, CD73, and CD90 and negative expression of CD45, CD34, CD14 or CD11b, CD79*α* or CD19, and HLA class II, and (3) differentiation potential to mesenchymal lineages including osteocytes, adipocytes, and chondrocyte [7]. Unfortunately, the frequency of MSCs in BM is low; MSCs represent 0.001–0.01% of BM mononuclear cells or lower [8]. Although the optimal dosage of MSCs in therapeutic applications is still unclear and will be dependent upon the type of therapy, 1.0–2.0 × 10^6^ MSCs per kg body weight is generally used [8]. Direct collection of such a large number of MSCs from BM is not practical. Therefore, it is necessary to expand isolated MSCs *in vitro* to obtain a sufficient number for therapeutic approaches.

MSCs have a rapid proliferation ability, achieving a thousandfold expansion of cell number in a two- to three- week period. However, inappropriate expansion may reduce the quality of MSCs. It is known that extensive *in vitro* culture induces cellular senescence that is associated with growth arrest and apoptosis [9]. In addition, particular therapeutic properties of MSCs may be lost during prolonged culture; for example, the cardioprotective effect of passage 5 MSCs is significantly reduced compared to passage 3 MSCs [10]. However, protocols of MSC preparation used in clinical studies remain inconsistent and suboptimal. There are surprisingly different protocols used in current clinical studies, in terms of culture materials (flasks, culture media, and supplements), seeding density, passaging, and storage. These factors can influence the important properties of MSCs, leading to reduced or unexpected therapeutic results [11]. In addition, such inconsistent protocols make comparison of the results between clinical studies difficult.

Establishment of optimal, standardized protocols for MSC isolation and expansion will therefore be a key for MSC-based therapies to become widespread, generic approaches. For this aim, understanding of currently used protocols with their scientific justification is essential. We hereby carefully searched the protocols used in recent clinical trials of MSC-based therapy by referring PubMed. As a result, a total of 47 reports, which sufficiently describe MSC-preparation methods, were found, published from January 2007 onwards (see Supplementary Table 1 in Supplementary Material available online at http://dx.doi.org/10.1155/2014/951512). This review article summarizes the information obtained from these clinical trials with further referencing to relevant experimental studies, highlighting issues and solutions associated with current protocols of MSC isolation and expansion.

## 2. Background of Clinical Trials of MSC-Based Therapy Analyzed in This Review

**Figure 1:**
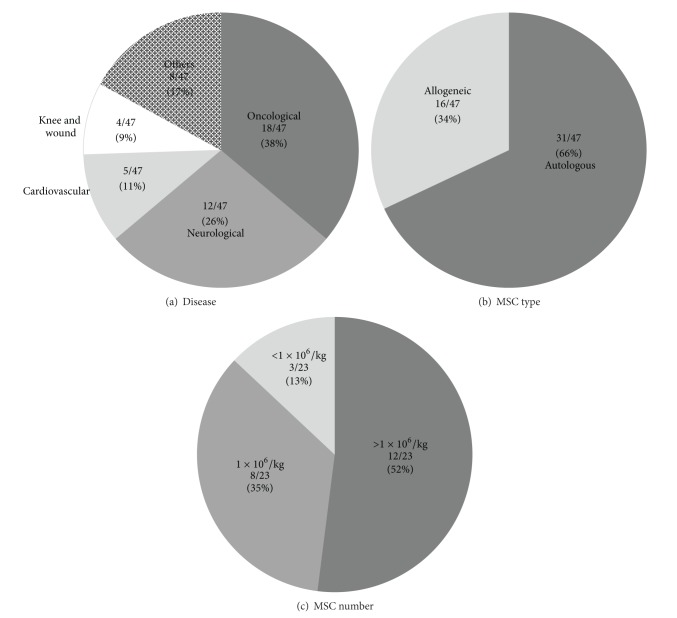
Background of the clinical trials of MSC-therapy reviewed in this article. A total of 47 clinical trials that have reported sufficient information on the MSC preparation were selected to review in this article. (a) A wide range of diseases were targeted by MSC therapy. (b) Both autologous and allogeneic MSCs were used for MSC therapy. (c) The number of MSCs administered was 1 × 10^6^/kg body weight or more in the majority of clinical trials. Some trials repeated the injection. See Supplementary Table 1 as well.

By literature search using PubMed, we selected 47 reports of clinical trials of BM-derived MSC-based therapy published between January 2007 and June 2013, which sufficiently describe the methods of isolation and expansion of MSCs (Supplementary Table 1). Most reports provide parts (not all) of the methodological information of interest to us. The trials aimed to treat a range of diseases, including oncological diseases (38%), followed by neurological diseases (26%) and cardiovascular diseases (11%) (Figure 1(a)). 66% of the studies used autologous MSCs, while the remaining 34% used allogeneic MSCs (Figure 1(b)). The number of MSCs injected ranged from 0.34 to 2.3 × 10^6^ cells/kg body weight; the majority of the reports administered 1-2 × 10^6^/kg body weight MSCs (Figure 1(c) and Supplementary Table 1). All these trials successfully supported feasibility to obtain the aimed number of MSCs, but with using a variety of isolation and expansion protocols. Furthermore, regardless of the protocols to prepare MSCs and cell number injected, no major safety issues that were directly caused by MSC, were reported.

## 3. Isolation of MSC from BM

### 3.1. BM Preparation for MSC Isolation

**Figure 2:**
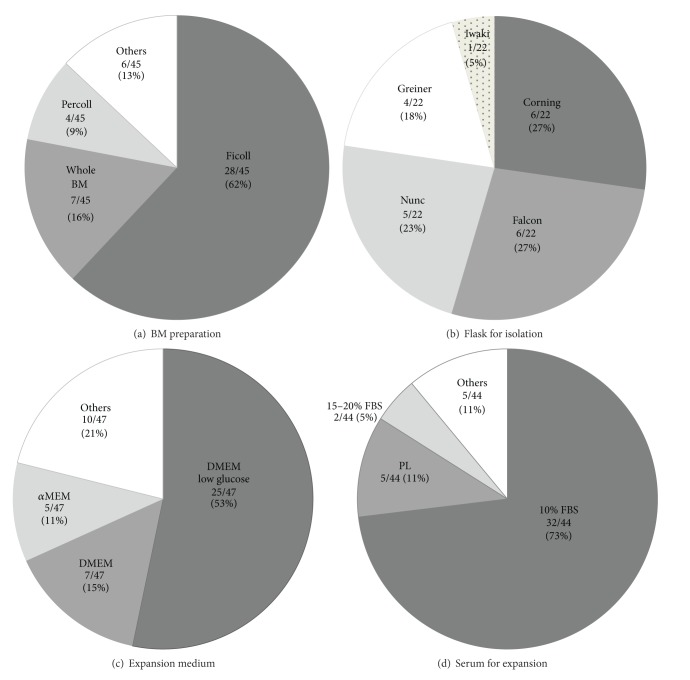
Protocols and materials used for MSC isolation and expansion. Different methods and materials were used in the recent MSC-therapy clinical trials, in terms of method for bone marrow (BM) preparation for MSC isolation (a), culture flask used for MSC isolation (b), culture medium used for MSCs expansion (c), and serum used for MSC expansion (d). PL: platelet lysate; FBS: fetal bovine serum.

Possible techniques to isolate MSCs from BM materials include cell adherence-based methods and cell-sorting methods, with the vast majority of previous clinical trials using the former method. The latter including fluorescence-activated cell sorting and immune-magnetic bead cell sorting [12] has the advantage of collecting a more purified MSC population. However, they are hardly used in clinical trials because of the lack of appropriately specific simple surface markers for MSCs, possible cellular damage, more expensive cost, and more demanding labor. For the adherence-based methods, either whole BM cells or BM mononuclear cells separated by density gradient centrifugation were used. The use of whole BM cells is clearly easier and yields higher numbers of adhered cells on plastic dishes with reduced loss of MSCs compared to density gradient separation methods. However, cells collected by an adherence method represent a heterogeneous mixture of cells, including not only MSCs but also hematopoietic cells at different differentiation/commitment stages, endothelial cells and endothelial progenitor cells. Although many of these contaminating cells may be removed during passaging, such contamination would affect the expansion of MSCs as well as the overall effect of the therapy. In order to isolate a more homogeneous initial MSC population, BM mononuclear cells can be separated from whole BM cells by density gradient centrifugation using either Ficoll (Paque, Hypaque, or Paque Premium) or Percoll (both available from GE Healthcare, Uppsala, Sweden). In the current studies we have analyzed, 62% used Ficoll-based density gradient separation, 16% used whole BM cells without separation, and another 9% employed Percoll-based density gradient separation (Figure 2(a)).

Percoll and Ficoll have usually been used at densities of 1.073 g/mL [13] and 1.077 g/mL [14], respectively, to isolate MSCs with high proliferative and differentiative potential. Percoll is a suspension of colloidal silica particles (diameter 15–30 nm), which has been widely used for separating cells, organelles, viruses, and other subcellular particles in basic science experiments, but it is not produced as a good manufacturing practice (GMP) grade reagent. Ficoll, a polymer of sucrose with a high synthetic molecular weight, is generated at GMP grade and has been frequently used for separating mononuclear cells and lymphocytes from peripheral blood in clinical practice for several decades, indicating clinical safety of the reagent. Recently Mareschi et al. compared MSCs collected via Percoll-separated mononuclear cells, Ficoll-separated mononuclear cells, and whole BM cells and found no significant differences in terms of gross morphology, differentiation potential, or immunophenotype between the collected cells [15]. However, the whole BM cell method apparently resulted in a greater Colony-Forming Unit-Fibroblast (CFU-F) number and improved cellular growth compared to gradient-separated cell methods. Given the other advantages in being less demanding in cost and labor, it is proposed that the whole BM cell method would be the first-choice method for MSC isolation from BM samples.

### 3.2. Flask for MSCs Isolation

There are many manufacturers that produce plastic flasks suitable for MSC isolation by the adherence-based method including Corning, Falcon, Nunc, and Greiner. Sotiropoulou et al. compared the effect of these 4 culture flasks to adhere MSCs [16] and indicated that greater numbers of MSCs were acquired in Corning flasks followed by Falcon, Nunc, and Greiner at 7 days after plating (without passaging). All these types of flasks are produced from polystyrene permanently rendered hydrophilic with corona discharge, using high voltage to create a reactive gas plasma [17]. This process for Falcon flasks takes place in a closed chamber, thus creating a consistent treatment surface. On the other hand, during manufacturing of the flasks from other companies, the gas is exposed to ambient air and therefore subjected to day-to-day environmental changes. In the real world, the most commonly used flask was Corning (27%) and Falcon (27%) equally, followed by Nunc (23%), Greiner (18%), and Iwaki (5%) in our analysis of current clinical trials (Figure 2(b)).

### 3.3. Cell Seeding Density for MSC Isolation

**Figure 3:**
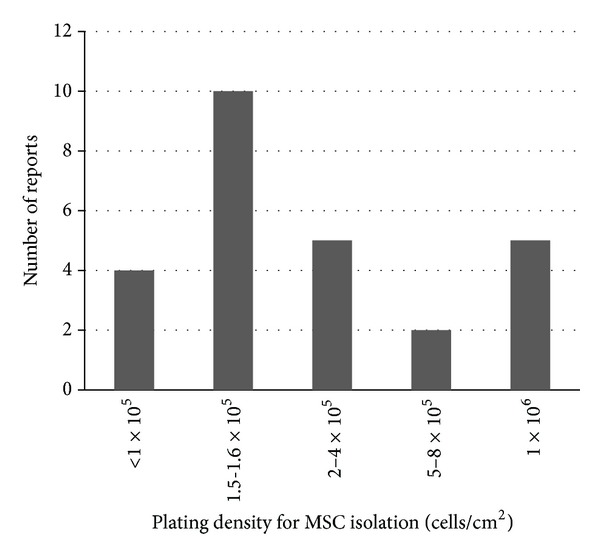
Plating density for MSC isolation. Plating cell densities of BM mononuclear cells for MSC isolation used in 26 clinical trial reports are presented. 1.5-1.6 × 10^5^ cells/cm^2^ was most frequently used.

Cell seeding density of BM mononuclear cells or whole BM cells is another important factor to determine the efficiency of MSC yield as this affects adherence of MSCs, contamination by other cell types, and initial growth of adhered MSCs. Sotiropoulou et al. reported that, between the range from 1 × 10^3^ to 2 × 10^5^ BM mononuclear cells/cm^2^, the lower initial seeding densities achieved increasingly larger numbers of adherent cells at Passage 0 [16]. Both et al. also reported that MSCs seeded at lower densities had a faster proliferation than those seeded at higher densities, with MSCs plated at 100 cells/cm^2^ reaching their target of 2 × 10^8^ cells 4 days faster than cells that were seeded at 5 × 10^3^ cells/cm^2^ [18]. Further decrease in the seeding density below 100 cell/cm^2^ showed a further increase in proliferation rate; however, there is a lower limit in the plating density in the clinical settings. Given that 1 × 10^7^–1 × 10^8^ BM mononuclear cells or a larger number of whole BM cells are commonly obtained, it is not practical to seed such a large number of cells at below 1 × 10^4^. Seeding 1 × 10^8^ BM mononuclear cells at 1 × 10^3^ cells/cm^2^ would require approximately 600 × 175 cm^2^ flasks, which is too high a cost in terms of materials and labor. As a matter of fact, the cell seeding density used in current clinical trials is quite high, with extreme variability ranging from 1.1 × 10^3^ to 1.0 × 10^6^ mononuclear cells/cm^2^ (Supplementary Table 1). The most commonly used seeding density of BM mononuclear cells is 1.5-1.6 × 10^5^ cells/cm^2^, followed by 1.0 × 10^6^ cells/cm^2^ and 2–4 × 10^5^ cells/cm^2^ (Figure 3). For the future clinical application, it is suggested that BM-mononuclear cells should be plated at as a low density as far as the cost, facility, and labor allow. There is very limited experimental evidence to discuss the optimal plating dose of whole BM cells but a pre-clinical study has shown that 10,000 cells/cm^2^ would be the most advantageous condition [15].

### 3.4. Medium and Supplement

Optimal medium and culture supplements for MSC isolation remain much less unstudied, compared to those for MSC expansion (see Section 4 for detailed information). In the vast majority of current clinical trials, the same medium and serum/supplement appeared to be simply used for both MSC isolation and expansion (very few reports described this particular method). This may be convenient and economical in practice; however, the most effective conditions for isolation and expansion of MSCs could be different, requiring further research to elucidate the optimal culture medium for MSC isolation.

## 4. Expansion of MSCs

### 4.1. Flask for MSCs Expansion

A comprehensive laboratory investigation of the proliferation efficacy of MSCs cultured on 4 major types of culture flasks has indicated that the most improved expansion of cultured MSCs was acquired in the flasks from Falcon, followed by those from Corning, Nunc, and Greiner, although the quality and functions of produced MSCs did not differ among the different types of flasks examined [16]. In contrast, the most commonly used flask in previous clinical trials we investigated here was Corning flasks (35%), followed by Nunc flasks (25%), Falcon flasks (20%), and Greiner flasks (20%) (Supplementary Table 1). In the majority of the reports, we found that the same manufacturer's flasks are preferably used for both isolation and expansion of MSCs. The use of the same manufacturer's flasks may be more convenient and economical; however, it should be noted that the optimal flask surface for initial isolation of MSCs could be different from that for MSC expansion as the scientific evidence indicates [16].

A wide surface area is required to obtain a sufficient number of MSCs for clinical application. To reduce the number of culture flasks used, manufacturing companies such as Nunc and Corning offer large, multilayered culture systems that can fit to usual cell culture incubators. Decreasing the number of flasks will improve the microbiological safety and traceability and also reduce staff workload and cost. The CellStacks (Corning, USA) and CellFactory (Nunc, Denmark) systems, which start from a unit surface area of 635 cm^2^, offer the possibility of 2, 5, 10, and 40 stages per container. In addition, these devices can be connected by tubes, allowing for convenient, sterile, GMP-compliant operations (e.g., culture initiation, medium exchange, and cell harvesting).

### 4.2. Basal Culture Medium

Basal culture medium consists of amino acids, glucose, and ions including calcium, magnesium, potassium, sodium, and phosphate. There is no doubt that the types of culture medium used affect proliferation and differentiation of MSCs. There is a preclinical report showing that DMEM is preferable to IMDM (Iscove's modified Dulbecco's medium) with respect to preservation of MSC stemness [19]. It has also been experimentally demonstrated that *α*MEM (minimal essential medium) better preserves osteogenic properties of MSCs and achieves higher CFU-F retrieval than DMEM [20].  Figure 2(c) shows that the basal culture media used for MSC expansion in current clinical trial includes DMEM-low glucose (53%), DMEM (15%), and *α*MEM (11%).

L-Glutamine is an essential nutrient for energy production as well as protein and nucleic acid synthesis in cell culture, and thus this is commonly supplemented into culture media. However, this spontaneously degrades in culture media, and its chemical breakdown and cellular metabolism lead to ammonia formation, possibly inhibiting cell growth [21]. To solve this issue, Glutamax is recently used as substitute for L-glutamine, as this is more stable in aqueous solutions and does not spontaneously degrade. Sotiropoulou et al. systemically compared the expansion efficacy of MSCs among 8 different basal media (IMDM, Optimem, *α*MEM with L-glutamine, *α*MEM with Glutamax, DMEM with low glucose and L-glutamine, DMEM with low glucose and Glutamax, DMEM with high glucose and L-glutamine, and DMEM with high glucose and Glutamax) [16]. The authors have found significant differences: among the 8 types of medium studied, *α*MEM containing Glutamax achieved the greatest expansion of cultured MSCs, followed by *α*MEM containing L-glutamine. Unfortunately many previous clinical trial papers did not clearly describe the type of glutamine used (Supplementary Table 1).

### 4.3. Growth Factor Supplement for MSC Expansion

It is known that growth factor supplement to culture medium enhances proliferation with maintenance of important properties of MSCs. In particular, fibroblast growth factor-2 (FGF2) [22], platelet-derived growth factor (PDGF) [23], epidermal growth factor (EGF) [24], transforming growth factor (TGF)-*β* [23], and insulin-like growth factor (IGF) [25, 26] play a role. Previously, fetal bovine serum (FBS) has been most frequently used (10% FBS in 73% and 15–20% FBS in 5%) to supply growth factors to MSC culture medium (Figure 2(d)), because FBS contains all these factors and is relatively readily available at clinical grade. However, it should be noted that FBS shows considerable variation in growth factor activity from batch to batch, and therefore large amounts of batch-tested FBS will need to be reserved for a clinical application. Furthermore, FBS remains associated with safety issues including transmission of prion or viral disease, anaphylatoxic reactions, and production of anti-FBS antibodies [27, 28]. Regulatory authorities in an increasing number of countries, including Paul-Ehrlich-Institute in Germany, now prohibit the clinical use of FBS, while in contrast the Australian Therapeutic Goods Authority allows the use of FBS for the production of clinical grade materials as long as it is sourced from cattle in a country free of bovine spongiform encephalitis such as Australia or New Zealand.

To avoid such a risk related to the use of animal materials, the use of human products, including human serum and platelet lysate, has been proposed. The effect of human allogeneic serum from adult donors to enhance proliferation of MSCs with preservation of important cellular properties is controversial [29, 30]. On the other hand, it will be problematic to acquire a large amount of autologous serum sufficient to generate clinically relevant numbers of MSCs. Moreover, autologous serum from elderly patients may have deteriorated capacity to support cell growth. Allogeneic human serum from umbilical cord blood [31] and placenta [32] has also been proposed as a potential alternative to replace FBS because these primitive tissues are a rich source of growth factors.

Platelet lysate has recently been a more preferred human product; more than 10% of previous clinical trials between 2007 and 2013 used Platelet lysate (Figure 2(d)). Platelet lysate can be easily obtained from apheresis products [26], as well as from buffy coats [33] of healthy volunteers. Immediately after collection, platelet products are frozen at −80°C and subsequently thawed to obtain the release of growth factors included in platelets with centrifugation to eliminate platelet bodies. The obtained growth factors include PDGFs, b-FGF, VEGF, IGF-1, and TGF-*β* [25, 26], which improve proliferative capacity of MSCs. Platelet lysate from several healthy donors may be pooled for the use [26]. Doucet et al. first demonstrated that growth factors contained in platelet lysate are able to promote MSC expansion in a dose-dependent manner [25]. This was further substantiated by the data showing that a culture medium supplemented with 5% platelet lysate is superior to 10% FBS in clonogenic efficiency and proliferative capacity of MSCs, therefore providing more efficient expansion, together with a significant time saving [26]. However, several studies have shown limitations of platelet lysate, including reduction of osteogenic or adipogenic differentiation potential [33, 34] and decreased immunosuppressive capacity with altered surface marker expression [35]. In addition, there is a risk that any allogeneic human product may be contaminated with human pathogens that might not be detected by routine screening. Moreover, crude blood derivatives are poorly defined and also suffer from batch-to-batch variation, and thus their ability to maintain MSC growth and therapeutic potentials could be variable. Further studies are need for platelet lysate to be part of a standard protocol.

Issues associated with human products encourage the use of serum-free and animal component-free MSC culture media. StemPro MSC SFM from Invitrogen is the first FDA-approved commercial product of this type. Agata et al. showed an enhanced effect of StemPro MSC SFM to improve rapid proliferation at early (<5) passage stages compared to FBS [36]. Of note, important characteristics of MSCs, including surface antigen expression, stemness, and differentiation potential, are different between MSCs cultured with FBS and MSCs with serum-free medium [36]. Although the formulations of these commercial media are not disclosed, it is important to evaluate each commercial media and select the most suitable product for each type of treatment.

### 4.4. Direct Addition of Growth Factors for MSC Culture

Although ideal growth factor supplements for MSC culture are still undefined, administration of several types of growth factors with or without serum or platelet lysate has been tested if it could increase MSC expansion with maintenance of important cellular properties. These include at least b-FGF [22], PDGF [23], TGF-*β*, EGF [24], and IGF1 [25, 26]. FGF2 induces excellent expansion efficiency of MSCs with maintenance of their differentiation potential and has been used for clinical trials of MSC-based therapy [37]. However, recent data suggests that b-FGF upregulates HLA-DR and Stro-1 and downregulates CD44 in a dose-dependent fashion [16, 38]. PDGFs were first found in platelets and they might be responsible for some of the platelet lysate activity in MSC growth. PDGFs have a role in osteogenic, adipogenic, and chondrogenic differentiation of MSCs; however, the primary effect is likely to be mitogenic action with inhibition of differentiation [39]. The PDGF-BB isoform can activate all PDGF receptors and therefore may be the best choice as a culture supplement. A recent report found that a combination of b-FGF, PDGF, and TGF-**β** could replace the serum component in cell culture medium to expand human MSCs without compromising differentiation potential, at least up to 5 passages [23]. Further evidence is required for this promising approach to become a widespread protocol.

### 4.5. Cell Plating Density for MSCs Expansion

Plating cell density is influential not only on initial isolation but on expansion of cultured MSCs. Generally, a higher plating density results in a reduced proliferation ability, probably due to contact inhibition and/or less availability of nutrients per cell [40]. The log phase lasts for a longer duration in cells plated at lower densities, and hence more population doublings occur due to a longer exponential growth phase [41]. A comparable study showed that, after 10 days in culture, BM-derived MSCs seeded at 2,500, 250, 25, and 2.5 cells/cm^2^ resulted in 2.7 ± 0.5, 4.8 ± 0.4, 6.7 ± 0.5, and 7.6 ± 1.0 population doublings [42]. This study also showed that the seeding density does not affect cellular properties of MSCs including cell surface marker expression.

**Figure 4:**
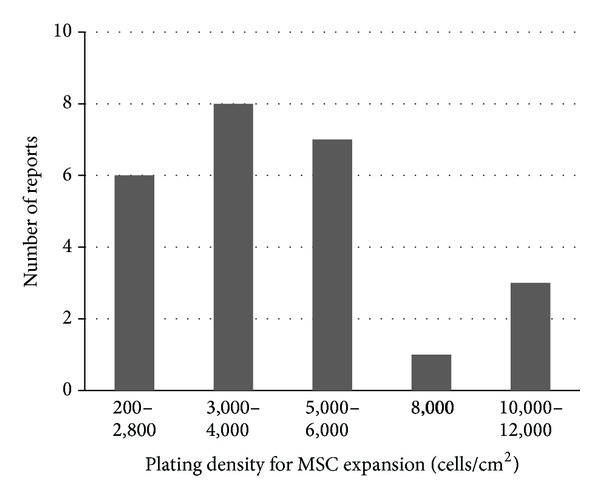
Plating density for MSC expansion. Plating cell densities for MSC expansion used in 25 clinical trial reports are presented. 3,000–6,000 cells/cm^2^ was commonly used.

However, for clinical-scale production (1 × 10^6^/kg body weight or more) of MSCs, use of a very low plating density is unrealistic due to demanding cost, facility, and labor; a plating density of 1,000 cells/cm^2^ is suggested as a reasonable, evidence-based compromise [43]. In the clinical arena, however, more compromise for the cost/labor is usually taken; over 75% of current clinical trials used plating densities of over 3,000 cells/cm^2^ (Figure 4).

## 5. Passaging and Storing of MSC

### 5.1. Dissociation of Adherent MSCs

**Figure 5:**
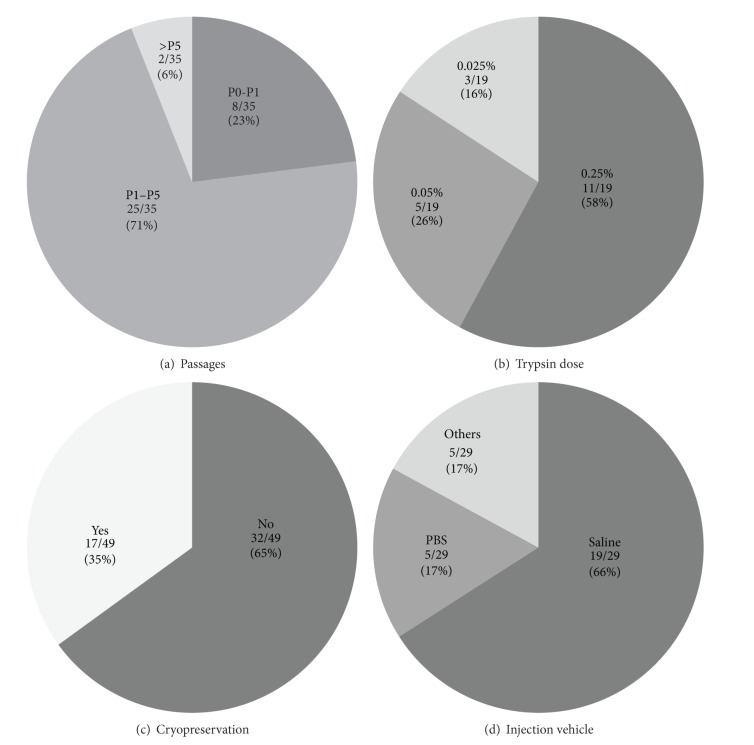
Preparations of MSCs. (a) Over 70% clinical trials used MSCs that received 1–5 passages. (b) Doses of trypsin used for passaging were widely varied. (c) Cryopreserved MSCs were used in 35% of clinical trials reviewed. In 47 clinical trials studied, two trials used both fresh cells and cryopreserved cells, thus the total number of reports shown in the graph is 49 (see Supplementary Table 1). (d) It was common to use saline as injection vehicle.

For the purpose of passaging for expansion or collection for administration, adhered MSCs on plastic flasks need to be dissociated. In our search, administered MSCs were received in less than 1 passage in 23%, 1–5 passages in 71%, and over 5 passages in 6% of reported clinical trials (Figure 5(a)). To this end, the majority of current clinical trials used enzymatic digestion using trypsin-EDTA solution (Supplementary Table 1). Of note, the concentration of trypsin-EDTA used was widely ranging; 0.25, 0.05, and 0.025% trypsin-EDTA was used in 58, 26, and 16% of previous trials, respectively (Figure 5(b)). Excessive trypsinisation can damage cells, while on the other hand insufficient trypsin-treatment will reduce the yield of cells. Optimal trypsinisation condition may be different among flask types used and cell density/confluence. Thus, the choice of trypsinisation conditions (not only concentration but also duration and temperature) should be carefully decided case-by-case based on scientific evidence. In addition, many previous trials appeared to utilise porcine-derived trypsin, which should be replaced with human trypsin or other alternatives [44, 45] to reduce safety concerns [46].

### 5.2. Storing of MSCs

Isolated and expanded BM-derived MSCs were sometimes stored until the time of treatment.

In our search 17 out of 49 (35%) trials used cryopreserved MSCs (Figure 5(c); two out of 47 clinical trials used both fresh cells and cryopreserved cells, making the total number 49). This allows for great flexibility in the clinical setting, but extreme caution is needed on possible adverse effects on MSCs. Although there are many preclinical studies showing that cryopreservation does not change the biological behavior of MSCs such as differentiation, growth, and/or surface marker expression [47, 48], on the other hand, there are reports warning hazardous effects by cryopreservation [49]. Further refinement of the protocol is warrantied. Important ingredients in current freezing solution include dimethylsulfoxide (DMSO) and serum. DMSO has been extensively used at 5–10% as a cryoprotectant with its high membrane permeability. However, DMSO can be damaging to cells when used in high concentration, especially during the thawing procedure. Also, if it remains in MSC suspension for administration, DMSO can cause adverse reactions in patients, including nausea, vomiting, tachycardia, bradycardia, and hypotension. Haack-Sørensen et al. [46, 50] advocate the use of 5% concentrations of DMSO together with 95% FBS. However, the use of animal sera will have a risk in the use for patients as discussed above. Defined, serum-free and animal component-free freezing media, such as Cryostor CS10 StemCell Technologies [51] or Plasmalyte-A [52], may be possible alternatives. Cell concentration during cryopreservation was proposed to be optimal with 0.5–1 × 10^6^/mL [53]. A controlled rate freezing method (freezing at rate of 1°C per minute) will achieve superior outcome than uncontrolled freezing [54].

### 5.3. Injection Vehicle

It is important to optimise the vehicle of MSCs for injection, as this will affect donor cell viability and loss before and after injection. In previous clinical trials, 66% used saline and 17% used PBS (Figure 5(d)). There were attempts to supplement human serum albumin to protect cells from environmental stress and prevent adherence to the walls of tubes and needles. Further systematic comparisons between injection vehicles are warrantied.

## 6. Conclusion and Future Perspective

Recent advance in basic and medical science and technologies has realised the employment of BM-derived MSCs for a variety of therapeutic indications including regenerative therapies. A sufficient sum of initial clinical trials has shown feasibility and safety of this approach at least and also suggested the therapeutic effect (though preliminary), encouraging further study for this approach to become an established generic treatment. One of the major hurdles for this development will be the establishment of optimised and standardized GMP-compliant protocols for isolation and expansion of MSCs. This review demonstrates how various the current protocols were. Many protocols lack scientific validation and appear to be suboptimal.

It is now urgently important to solve this issue of the lack of conformity between MSC manufacturing protocols, which is considered as potential threat to further development of MSC-based therapy. As summarised in this review, a range of relevant scientific evidence is available for this purpose. Active cooperation between academics, clinicians, companies, and regulatory authorities is encouraged in order to develop international standards for BM-derived MSC production, which should be evidence-based, regulatory authority-compliant, of good medical practice grade, cost-effective, and clinically practical, for the future success of MSC-based therapy.

## Supplementary Material

MSC-preparation protocols used in 47 recent clinical trials of MSC-based therapy, published from January 2007 onwards, are summarised in Supplementary Table 1. References used in Supplementary Table 1 are found in Supplementary References.
