# Supplementary material for: Mesenchymal Stem Cells for Regenerative Therapy: Optimization of Cell Preparation Protocols
Source: Biomed Res Int. 2014 Jan 6;2014:951512. doi: 10.1155/2014/951512 (PMC3912818; doi:10.1155/2014/951512)
Supplement: Supplementary file 2 [file 951512.f2.pdf]

Supplementary References

[55] V. Falanga, S. Iwamoto, M. Chartier *et al.*, "Autologous bone marrow-derived cultured mesenchymal stem cells delivered in a fibrin spray accelerate healing in murine and human cutaneous wounds," *Tissue Engineering*, vol. 13, no. 6, pp. 1299-1312, 2007.

[56] L. M. Ball, M. E. Bernardo, H. Roelofs *et al.*, "Cotransplantation of ex vivo expanded mesenchymal stem cells accelerates lymphocyte recovery and may reduce the risk of graft failure in haploidentical hematopoietic stem-cell transplantation," *Blood*, vol. 110, no. 7, pp. 2764-2767, 2007.

[57] M. Mohyeddin-Bonab, M. R. Mohamad-Hassani, K. Alimoghaddam *et al.*, "Autologous in vitro expanded mesenchymal stem cell therapy for human old myocardial infarction," *Archives of Iranian medicine*, vol. 10, no. 4, pp. 467-473, 2007.

[58] D. G. Katritsis, P. Sotiropoulou, E. Giazitzoglou, E. Karvouni, and M. Papamichail, "Electrophysiological effects of intracoronary transplantation of autologous mesenchymal and endothelial progenitor cells," *Europace*, vol. 9, no. 3, pp. 167-171, 2007.

[59] M. Mohyeddin Bonab, S. Yazdanbakhsh, J. Lotfi *et al.*, "Does mesenchymal stem cell therapy help multiple sclerosis patients? Report of a pilot study," *Iranian Journal of Immunology*, vol. 4, no. 1, pp. 50-57, 2007.

[60] M. Mohamadnejad, K. Alimoghaddam, M. Mohyeddin-Bonab *et al.*, "Phase 1 trial of autologous bone marrow mesenchymal stem cell transplantation in patients with decompensated liver cirrhosis," *Archives of Iranian medicine*, vol. 10, no. 4, pp. 459-466, 2007.

[61] O. Ringden, M. Uzunel, B. Sundberg *et al.*, "Tissue repair using allogeneic mesenchymal stem cells for hemorrhagic cystitis, pneumomediastinum and perforated colon," *Leukemia*, vol. 21, no. 11, pp. 2271-2276, 2007.

[62] P. H. Lee, J. W. Kim, O. Y. Bang, Y. H. Ahn, I. S. Joo, and K. Huh, "Autologous mesenchymal stem cell therapy delays the progression of neurological deficits in patients with multiple system atrophy," *Clinical Pharmacology and Therapeutics*, vol. 83, no. 5, pp. 723-730, 2008.

[63] I. Muller, S. Kordowich, C. Holzwarth *et al.*, "Application of multipotent mesenchymal stromal cells in pediatric patients following allogeneic stem cell transplantation," *Blood Cells, Molecules and Diseases*, vol. 40, no. 1, pp. 25-32, 2008.

[64] K. Le Blanc, F. Frassoni, L. Ball *et al.*, "Mesenchymal stem cells for treatment of steroid-resistant, severe, acute graft-versus-host disease: a phase II study," *Lancet*, vol. 371, no. 9624, pp. 1579-1586, 2008.

[65] H. Ning, F. Yang, M. Jiang *et al.*, "The correlation between cotransplantation of mesenchymal stem cells and higher recurrence rate in hematologic malignancy patients: outcome of a pilot clinical study," *Leukemia*, vol. 22, no. 3, pp. 593-599, 2008.

[66] L. Mazzini, K. Mareschi, I. Ferrero *et al.*, "Stem cell treatment in Amyotrophic Lateral Sclerosis," *Journal of the Neurological Sciences*, vol. 265, no. 1-2, pp. 78-83, 2008.

[67] C. J. Centeno, D. Busse, J. Kisiday, C. Keohan, M. Freeman, and D. Karli, "Increased knee cartilage volume in degenerative joint disease using percutaneously implanted, autologous mesenchymal stem cells," *Pain Physician*, vol. 11, no. 3, pp. 343-353, 2008.

[68] L. Sun, K. Akiyama, H. Zhang *et al.*, "Mesenchymal stem cell transplantation reverses multiorgan dysfunction in systemic lupus erythematosus mice and humans," *Stem Cells*, vol. 27, no. 6, pp. 1421-1432, 2009.

[69] M. von Bonin, F. Stolzel, A. Goedecke *et al.*, "Treatment of refractory acute GVHD with third-party MSC expanded in platelet lysate-containing medium," *Bone Marrow Transplantation*, vol. 43, no. 3, pp. 245-251, 2009.

[70] L. V. Kursova, A. G. Konoplyannikov, V. V. Pasov, I. N. Ivanova, M. V. Poluektova, and O.A. Konoplyannikova, “Possibilities for the use of autologous mesenchymal stem cells in the therapy of radiation-induced lung injuries,” *Cell Technologies in Biology and Medicine,* no. 2 , pp. 542-546, 2009.

[71] N. Meuleman, T. Tondreau, I. Ahmad *et al.*, “Infusion of mesenchymal stromal cells can aid hematopoietic recovery following allogeneic hematopoietic stem cell myeloablative transplant: a pilot study,” *Stem Cells and Development,* vol. 18, no. 9, pp. 1247-1252, 2009.

[72] A. M. Haleem, A. A. Singergy, D. Sabry *et al.*, “The Clinical Use of Human Culture-Expanded Autologous Bone Marrow Mesenchymal Stem Cells Transplanted on Platelet-Rich Fibrin Glue in the Treatment of Articular Cartilage Defects: A Pilot Study and Preliminary Results,” *Cartilage,* vol. 1, no. 4, pp. 253-261, 2010.

[73] B. Yamout, R. Hourani, H. Salti *et al.*, “Bone marrow mesenchymal stem cell transplantation in patients with multiple sclerosis: a pilot study,” *Journal of Neuroimmunology,* vol. 227, no. 1-2, pp. 18518-9, 2010.

[74] D. Karussis, C. Karageorgiou, A. Vaknin-Dembinsky *et al.*, “Safety and immunological effects of mesenchymal stem cell transplantation in patients with multiple sclerosis and amyotrophic lateral sclerosis,” *Archives of Neurology,* vol. 67, no. 10, pp. 1187-1194, 2010.

[75] M. Duijvestein, A. C. Vos, H. Roelofs *et al.*, “Autologous bone marrow-derived mesenchymal stromal cell treatment for refractory luminal Crohn's disease: results of a phase I study,” *Gut,* vol. 59, no. 12, pp. 1662-1669, 2010.

[76] F. Carrion, E. Nova, C. Ruiz *et al.*, “Autologous mesenchymal stem cell treatment increased T regulatory cells with no effect on disease activity in two systemic lupus erythematosus patients,” *Lupus,* vol. 19, no. 3, pp. 317-322, 2010.

[77] F. Baron, C. Lechanteur, E. Willems *et al.*, “Cotransplantation of mesenchymal stem cells might prevent death from graft-versus-host disease (GVHD) without abrogating graft-versus-tumor effects after HLA-mismatched allogeneic transplantation following nonmyeloablative conditioning,” *Biology of Blood and Marrow Transplantation,* vol. 16, no. 6, pp. 838-847, 2010.

[78] J. Liang, H. Zhang, B. Hua *et al.*, “Allogenic mesenchymal stem cells transplantation in refractory systemic lupus erythematosus: a pilot clinical study,”*Annals of the Rheumatic Diseases,* vol. 69, no. 8, pp. 1423-1429, 2010.

[79] J. S. Lee, J. M. Hong, G. J. Moon *et al.*, “A long-term follow-up study of intravenous autologous mesenchymal stem cell transplantation in patients with ischemic stroke,” *Stem Cells,* vol. 28, no. 6, pp. 1099-1106, 2010.

[80] H. Zhou, M. Guo, C. Bian *et al.*, “Efficacy of bone marrow-derived mesenchymal stem cells in the treatment of sclerodermatous chronic graft-versus-host disease: clinical report,” *Biology of Blood and Marrow Transplantation,* vol. 16, no. 3, pp. 403-412, 2010.

[81] X. Zhang, J. Y. Li, K. Cao, *et al.*, “Cotransplantation of HLA-identical mesenchymal stem cells and hematopoietic stem cells in Chinese patients with hematologic diseases,” *International Journal of Laboratory Hematology,* vol. 32, no. 2, pp. 256-264, 2010.

[82] J. Y. Weng, X. Du, S. X. Geng *et al.*, “Mesenchymal stem cell as salvage treatment for refractory chronic GVHD,” *Bone Marrow Transplantation,* vol. 45, no. 12, pp. 1732-1740, 2010.

[83] Z. Yang, F. Zhang, W. Ma *et al.*, “A novel approach to transplanting bone marrow stem cells to repair human myocardial infarction: delivery via a noninfarct-relative artery,” *Cardiovascular Therapeutics,* vol. 28, no. 6, pp. 380-385, 2010.

[84] F. Davatchi, B. S. Abdollahi, M. Mohyeddin, F. Shahram, and B. Nikbin, "Mesenchymal stem cell therapy for knee osteoarthritis. Preliminary report of four patients," *International Journal of Rheumatic Diseases*, vol. 14, no. 2, pp. 211-215, 2011.
[85] G. P. Lasala, J. A. Silva, B. A. Kusnick, J. J. Minguell, "Combination stem cell therapy for the treatment of medically refractory coronary ischemia: a Phase I study," *Cardiovascular Revascularization Medicine,* vol. 12, no. 1, pp. 29-34, 2011.
[86] A. R. Williams, B. Trachtenberg, D. L. Velazquez *et al.*, "Intramyocardial stem cell injection in patients with ischemic cardiomyopathy: functional recovery and reverse remodeling," *Circulation Research*, vol. 108, no. 7, pp. 792-796, 2011.
[87] P. Connick, M. Kolappan, R. Patani *et al.*, "The mesenchymal stem cells in multiple sclerosis (MSCIMS) trial protocol and baseline cohort characteristics: an open-label pre-test: post-test study with blinded outcome assessments," *Trials,* vol. 12, pp. 62, 2011.
[88] R. Ciccocioppo, M. E. Bernardo, A. Sgarella *et al.*, "Autologous bone marrow-derived mesenchymal stromal cells in the treatment of fistulising Crohn's disease," *Gut,* vol. 60, no. 6, pp. 788-798, 2011.
[89] N. Perico, F. Casiraghi, M. Introna *et al.*, "Autologous mesenchymal stromal cells and kidney transplantation: a pilot study of safety and clinical feasibility," *Clinical Journal of the American Society of Nephrology,*vol. 6, no. 2, pp. 412-422, 2011.
[90] O. Honmou, K. Houkin, T. Matsunaga *et al.*, "Intravenous administration of auto serum-expanded autologous mesenchymal stem cells in stroke," *Brain,* vol. 134, no. Pt 6, pp. 1790-1807, 2011.
[91] D. Wang, H. Zhang, M. Cao *et al.*, "Efficacy of allogeneic mesenchymal stem cell transplantation in patients with drug-resistant polymyositis and dermatomyositis," ***Annals of the Rheumatic Diseases*, v**ol. 70, no. 7, pp. 1285-1288, 2011.
[92] J. Tan, W. Wu, X. Xu *et al.*, "Induction therapy with autologous mesenchymal stem cells in living-related kidney transplants: a randomized controlled trial," *The Journal of the American Medical Association,* vol. 307, no. 11, pp. 1169-1177, 2012.
[93] S. Karamouzian, S. N. Nematollahi-Mahani, N. Nakhaee, and H. Eskandary, "Clinical safety and primary efficacy of bone marrow mesenchymal cell transplantation in subacute spinal cord injured patients," *Clinical Neurology and Neurosurgery,* vol. 114, no. 7, pp. 935-939, 2012.
[94] L. A. Kuzmina, N. A. Petinati, E. N. Parovichnikova *et al.*, "Multipotent Mesenchymal Stromal Cells for the Prophylaxis of Acute Graft-versus-Host Disease-A Phase II Study," *Stem Cells International,* vol. 2012, pp. 968213, 2012.
[95] P. Connick, M. Kolappan, C. Crawley *et al.*, "Autologous mesenchymal stem cells for the treatment of secondary progressive multiple sclerosis: an open-label phase 2a proof-of-concept study," *The Lancet Neurology,* vol. 11, no. 2, pp. 150-156, 2012.
[96] R. Herrmann, M. Sturm, K. Shaw *et al.*, "Mesenchymal stromal cell therapy for steroid-refractory acute and chronic graft versus host disease: a phase 1 study," *International Journal of Hematology,* vol. 95, no. 2, pp. 182-188, 2012.
[97] M. El-Ansary, I. Abdel-Aziz, S. Mogawer *et al.*, "Phase II trial: undifferentiated versus differentiated autologous mesenchymal stem cells transplantation in Egyptian patients with HCV induced liver cirrhosis," *Stem Cell Reviews and Reports,* vol. 8, no. 3, pp. 972-981, 2012.
[98] P. H. Lee, J. E. Lee, H. S. Kim *et al.*, "A randomized trial of mesenchymal stem cells in multiple system atrophy," *Annals of Neurology,* vol. 72, no. 1, pp. 32-40, 2012.
[99] M. E. Reinders, J. W. de Fijter, H. Roelofs *et al.*, "Autologous bone marrow-derived mesenchymal stromal cells for the treatment of allograft rejection after renal

transplantation: results of a phase I study," *Stem Cells Translational Medicine*, vol. 2, no. 2, pp. 107-111, 2013.

[100] M. Mohamadnejad, K. Alimoghaddam, M. Bagheri *et al.*, "Randomized placebo-controlled trial of mesenchymal stem cell transplantation in decompensated cirrhosis," *Liver International*, pp. 1-7, 2013.
